# Femto-LASIK after Deep Anterior Lamellar Keratoplasty to Correct Residual Astigmatism: A Long-Term Case Series Study

**DOI:** 10.3390/medicina58081036

**Published:** 2022-08-02

**Authors:** Belén Alfonso-Bartolozzi, Carlos Lisa, Luis Fernández-Vega-Cueto, David Madrid-Costa, José F. Alfonso

**Affiliations:** 1 Fernández-Vega Ophthalmological Institute, 33012 Oviedo, Spain; belen.alfonso@fernandez-vega.com (B.A.-B.); carloslisa@fernandez-vega.com (C.L.); lfvc@fernandez-vega.com (L.F.-V.-C.); 2 Clinical and Experimental Eye Research Group (CEER), Faculty of Optics and Optometry, Universidad Complutense de Madrid, 28037 Madrid, Spain; damadrid@ucm.es

**Keywords:** Femto-LASIK, deep anterior lamellar keratoplasty, DALK

## Abstract

**Purpose:** To evaluate the long-term outcomes of femtosecond laser-assisted in situ keratomileusis (Femto-LASIK) to correct residual astigmatism after deep anterior lamellar keratoplasty (DALK). **Methods:** This retrospective case series study included 10 eyes that underwent Femto-LASIK after a DALK. The refractive error, uncorrected (UDVA) and corrected (CDVA) distance visual acuities, thinnest corneal thickness (TCT), and central corneal thickness (CCT) were registered. The postoperative follow-up ranged between 36 and 60 months. **Results:** All surgeries were uneventful, with no intra- or postoperative complications. The mean UDVA (Snellen scale) rose from 0.13 ± 0.05 to 0.47 ± 0.15 six months after Femto-LASIK (*p* < 0.001). All cases experienced a significant improvement in UDVA. None of the eyes lost lines of CDVA, and seven eyes (70%) improved the CDVA compared to preoperative values. The refractive cylinder changed from a preoperative value of −3.88 ± 1.00 D to −0.93 ± 0.39 six months after Femto-LASIK (*p* < 0.0001). In eight eyes (80%), the UDVA and refractive outcomes remained stable at postoperative follow-up visits. In contrast, one eye experienced a refractive regression over the follow-up. TCT and CCT were stable at the different postoperative follow-up visits. **Conclusions:** Our findings suggest that Femto-LASIK might safely and effectively corrects residual astigmatism after DALK. Despite these encouraging results, further long-term studies, including a larger number of cases, are required to confirm the safety of the procedure. The refractive stability in eyes with prior RK might be lower than for other DALK indications.

## 1. Introduction

Deep lamellar anterior keratoplasty (DALK) has significant advantages over penetrating keratoplasty (PKP), such as reducing the risk of endophthalmitis and minor loss of endothelial cell density (ECD), among others [1]. Consequently, DALK is becoming the preferred option for corneal transplantation in eyes in which the host endothelium is healthy and can be preserved [1]. However, DALK has no advantages for refractive error outcomes [1]. Therefore, similarly to PKP, DALK is associated with a significant refractive error postoperatively, even higher than that reported after PKP [2,3,4]. 

Anisometropia or high degrees of refractive error corrected with spectacles may not be well tolerated. Furthermore, patients may experience contact lens intolerance after DALK. These potential drawbacks of spectacles or contact lenses to correct the residual refractive error lead to a significant limitation in visual rehabilitation. 

In PKP, the use of excimer laser for postoperative refractive error correction has been widely studied [5,6,7,8,9,10,11,12,13,14,15,16,17,18,19,20,21,22,23,24]. However, in DALK, knowledge is limited to a few studies and did not report an extended follow-up [25,26,27,28]. An earlier report analyzed the clinical outcomes of femtosecond laser-assisted in situ keratomileusis (Femto-LASIK) to correct residual refractive error after DALK for keratoconus. The study found that Femto-LASIK safely and effectively reduced the refractive error after DALK, significantly improving the uncorrected (UDVA), and corrected distance visual acuity (CDVA) 6 months after the surgery. It would be interesting to analyze if these favourable outcomes remain over a longer follow-up. 

In the current study, a case series of 10 eyes that underwent Femto-LASIK after DALK for different indications were analyzed case-by-case over a follow-up of at least 36 months.

## 2. Material and Methods

This study is a long-term case series analysis of patients who underwent Femto-LASIK after DALK surgery at the Fernández-Vega Ophthalmological Institute in Oviedo, Spain. It was conducted in compliance with the tenets of the Declaration of Helsinki, and full ethical approval from the institute was obtained. After receiving a complete description of the nature and the possible consequences of surgery, all patients provided informed consent. 

Indications for DALK were keratoconus (n = 3), post-LASIK ectasia (n = 2), RK (n = 4), and corneal scar (n = 1). The same surgeon (J.F.A.) performed all DALK surgeries using the Anwar technique [29]. The time between the DALK procedure and Femto-LASIK to correct the residual refractive error was at least six months after complete sutures removal. Before the Femto-LASIK surgery, all the patients had a clear corneal graft, refractive stability for at least six months after all sutures were removed, endothelial cell density (ECD) greater than 1500 cell/mm^2^, and a minimum corneal thickness of 500 µm. None of the patients had ocular or systemic diseases with a potential impact on graft survival.

**Figure 1:**
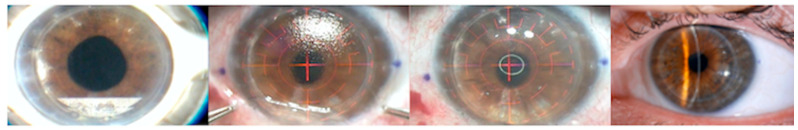
Sequence showing a Femto-LASIK surgery after DALK. The flap diameter was smaller than the graft diameter, and the excimer laser ablation was performed within the corneal graft limits without involving the graft-host junction.

The option of LASIK with femtosecond laser surgery was agreed upon between the surgeon and the patient after fully understanding the potential risks. Surgery was performed under topical anaesthesia using the IntraLase femtosecond laser FS60 (Advanced Medical Optics, Inc., Santa Ana, CA, USA) to create the flap and the VISX Star S4 (Advanced Medical Optics, Inc., Santa Ana, CA, USA) excimer laser to perform corneal photoablation. Femtosecond laser flaps were programmed with the following settings: 100-μm thickness; 7.0 to 8.5 mm diameter (0.1–0.2 mm smaller than graft diameter, without including the graft-host junction) (Figure 1); 55-degree superior hinge angle to achieve equivalent corneal stromal surface exposure; 60-degree side-cut angle; laser raster patterns spot and line separation of 7 and 7 μm; and stromal energy of 1.0 μJ with side-cut energy of 1.8 μJ. A pocket was created with the femtosecond laser to avoid an opaque bubble layer. The excimer laser was programmed with the conventional VISX S4 algorithm to correct the refractive cylinder (Advanced Medical Optics, Inc., Santa Ana, CA, USA). The postoperative treatment consisted of a regimen of 1% dexamethasone, and ciprofloxacin 0.3% drops 4 times a day for one week. Antibiotic drops were then discontinued, and dexamethasone was progressively tapered down over the next three weeks. Furthermore, plasma rich in growth factors (PRGF) eye drops were added topically four times daily for at least three months. The postoperative follow-up ranged between 36 and 60 months.

Manifest refraction, UDVA and CDVA were registered at each postoperative follow-up visit after the Femto-LASIK surgery. The manifest refraction was analyzed using the power vector method proposed by Thibos and Horner [30]. Furthermore, the following topographic and pachymetric parameters were reviewed (Sirius, CSO, Italy): keratometry, thinnest corneal thickness (TCT), and central corneal thickness (CCT).

Statistical analysis was carried out using SPSS software for Windows (version 15.0, SPSS, Inc., Chicago, IL, USA). Preoperative and postoperative data were compared using the Friedman test. A *p*-value less than 0.05 was considered statistically significant. Data are reported as the mean ± standard deviation (SD).

## 3. Results

The study included 10 eyes of 10 patients (mean age of 51.8 ± 8.6; range 45 to 74). The mean time between the DALK procedure and Femto-LASIK to correct the residual refractive error was 27.6 ± 4.8 months (range 21–33 months). All the Femto-LASIK surgeries were uneventful, with no intra- or postoperative complications. 

**Figure 2:**
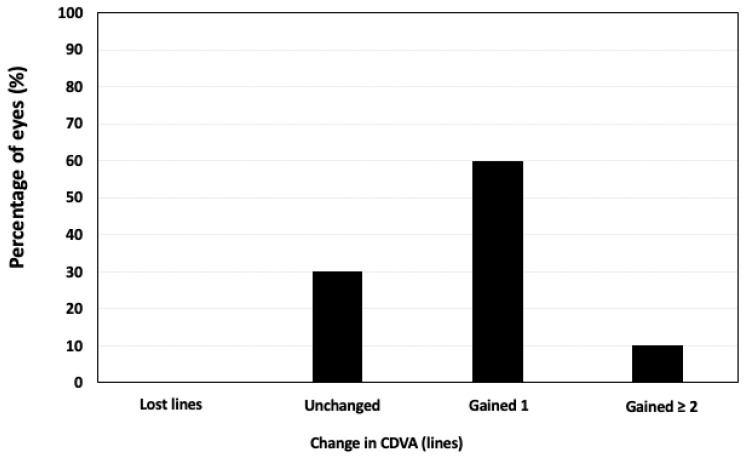
Variation in CDVA 6 months after Femto-LASIK.

Table 1 shows the UDVA, CDVA, and manifest refraction before Femto-LASIK and throughout the postoperative period for each case. The mean post-Femto-LASIK follow-up period was 48.0 ± 12.6 months (range 36–60 months). The mean UDVA (Snellen scale) rose from 0.13 ± 0.05 to 0.47 ± 0.15 at six months after Femto-LASIK (*p* < 0.001). All cases experienced a significant improvement in UDVA. None of the eyes lost lines of CDVA, and seven eyes (70%) improved the CDVA compared to preoperative values (Figure 2). 

**Figure 3:**
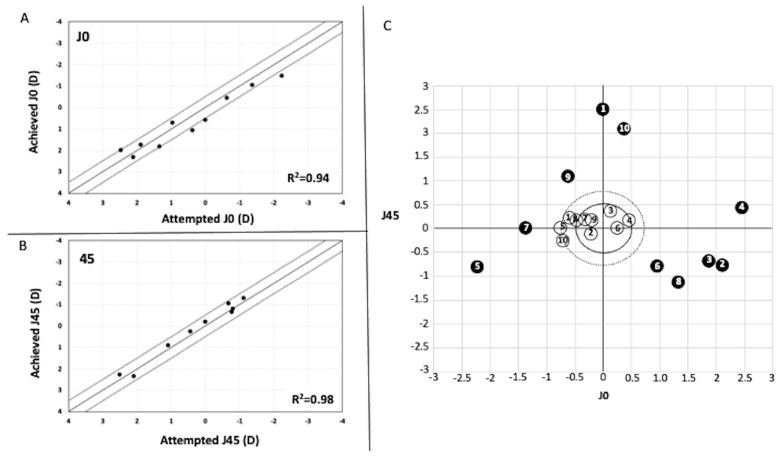
Plots showing the achieved against attempted correction (predictability) of the astigmatic components J0 (**A**) and J45 (**B**) of 6 months Femto-LASIK. (**C**) Representation of the astigmatic vector (J0 and J45) before surgery and 6 months after Femto-LASIK. (The area inside the dot-line circle represents a refractive cylinder ≤ −1.5 D, and the area inside the black-line circle a refractive cylinder ≤ −1.0 D).

The SE decreased from a preoperative value of −2.34 ± 2.03 to −0.99 ± 0.86 at six months after Femto-LASIK (*p* = 0.01). The refractive cylinder reduced from −3.88 ± 1.00 D preoperatively to −0.93 ± 0.39 six months after Femto-LASIK (*p* < 0.0001). Figure 3A,B shows the correlation analysis between the targeted versus achieved (predictability) of the astigmatism component (J0 and J45) six months after Femto-LASIK. A total of 7 eyes (70%) and 10 eyes (100%) were within ±0.50 D of the target refraction for J0 (r^2^ = 0.94) and J45 (r^2^ = 0.98), respectively. In all cases, the astigmatism components (J0 and J45) significantly moved towards the graph’s origin (0, 0) after Femto-LASIK (Figure 3C). In all cases, the preoperative refractive cylinder was ≥2.50 D (range −2.50 to −5.00). Conversely, postoperatively, the refractive cylinder was ≤1.50 D in all eyes (range −0.50 to −1.50 D) (Figure 3C, dot line circle). Seven eyes (70%) had a postoperative refractive cylinder ≤−1.0 D (Figure 3C black line circle). A bioptic surgery was planned in cases 2 and 4 (Femto-LASIK for cylinder correction, and then three months later, a spherical Implantable Collamer Lens (ICL; STAAR Surgical Inc., Monrovia, CA, USA) was implanted to correct the associated spherical component). 

Regarding stability, CDVA was stable over the follow-up period, and none of the eyes lost lines of CDVA over the postoperative follow-up period (Table 1). In eight eyes (80%; cases 1, 2, 3, 4, 5, 6, 8, and 10), the UDVA and refractive outcomes remained stable at postoperative follow-up visits (Table 1). In contrast, two eyes (cases 7 and 9; Table 1) experienced refractive changes over the follow-up and a decrease in UDVA. The K readings were stable over post-Femto-LASIK follow-up in all cases except case 9. 

The mean CCT decreased from 590 ± 44.25 µm preoperatively to 523 ± 45.55 µm at 6 months postoperatively (*p* = 0.0006), and then it remained unchanged over the follow-up (529.25 ± 41.75 µm at the last visit (*p* = 0.12)). The TCT was 509 ± 57.9 µm at 6 months after Femto-LASIK and 512 ± 50.9 µm at the last follow-up visit (*p* = 0.2). Preoperative mean ECD was 1782.9 ± 462.2 cell/mm^2^ and postoperatively was 1785.9 ± 336.7 cell/mm^2^ and 1749.2 ± 312.3 cell/mm^2^ at 6 months and the last follow-up visit, respectively (*p* = 0.4). 

## 4. Discussion 

In our study, the post-DALK (before Femto-LASIK) mean spherical equivalent was −2.34 ± 2.03D, the mean refractive cylinder −3.88 ± 1.00D, and the mean UDVA (Snellen scale) was 0.13 ± 0.05. These results agree with those previously reported [31,32]. These findings indicate that whether that refractive error is not well tolerated with spectacles or induces anisometropia, the DALK outcomes may not be as successful as expected due to the unsatisfactory postoperative UDVA. To overcome this, in the current study, we present 10 eyes that underwent Femto-LASIK surgery to correct residual astigmatism after a DALK procedure. 

The first issue that should be addressed is when planning the surgery. It has been reported that DALK provides stable long-term visual and refractive outcomes from 6 months after complete sutures removal [33,34]. Therefore, it is recommended to wait until this time to plan the post-DALK refractive error correction [33]. In the current study, all Femto-LASIK surgeries were carried out at least 6 months after complete sutures removal. The mean interval time between surgeries (DALK procedure and Femto-LASIK) was 26.6 ± 6.0 months (range 18–33 months). In our series of cases, the UDVA improved significantly in all eyes at 6 months of Femto-LASIK (ranged from 2 to 6 lines of UDVA). None of the eyes lost lines of CDVA, and seven eyes (70%) showed a gain of lines of CDVA. Only four previous studies have evaluated post-DALK residual refractive error correction with the excimer laser. Leccisotti [25] analyzed 10 eyes with PRK with Mitomycin C. Toru Acar et al. [26] reported 13 cases with microkeratome LASIK. Sorkin et al. [27] assessed 14 eyes treated with custom PRK to correct irregular astigmatism. Finally, Balestrazzi et al. [28] evaluated 13 eyes after Femto-LASIK. Our CDVA results were comparable with those previously found in these previous studies. The increase in UDVA was similar to that reported by Sorkin et al. [27] but significantly lower than those reported in the other three studies [25,26,28]. These differences are attributable to the target refractive error attempted to correct. In our study, similarly to that of Sorkin et al. [27], we focused on correcting astigmatism, whereas, in the other three, the aim was to achieve emmetropia. Consequently, in our study, the induced change in SE was around 1.25D, significantly lower than those previously reported (around 4.00 D [25,26,27]). In two cases (cases 2 and 4), a Bioptic procedure was planned, and a spherical ICL was implanted 3 months after Femto-LASIK to correct the sphere. In both cases, Femto-LASIK could have been programmed to achieve emmetropia. However, attempting to correct the full refractive error could have exposed these patients to poor visual quality. In cases 1 and 6, the fellow eye’s sphere was similar to the preoperative one in the treated eye. Therefore, the sphere was purposefully not corrected to prevent anisometropia. Analyzing the refractive cylinder’s attempted correction, we found a satisfactory correction rate, decreasing from a preoperative value of -3.88 ± 1.00 D to −0.93 ± 0.39 D six months after Femto-LASIK. All cases had a refractive cylinder ≥ 2.50 D preoperatively, whereas postoperatively, the refractive cylinder was ≤1.50 D in all eyes, and seven eyes (70%) had a postoperative refractive cylinder ≤ −1.00D. A good correlation between the attempted and achieved J0 and J45 astigmatism components was obtained (R^2^ = 0.94 and 0.98, respectively). We found that 70% and 100% of the eyes were within ±0.50 D of the intended J0 and J45 astigmatism components, respectively. 

It is essential to consider that the main aim of Femto-LASIK in post-DALK eyes should be anisometropia resolution and good visual acuity. Our findings show in post-DALK eyes that Femto-LASIK effectively reduced the refractive cylinder up to 5.00 D, improving both UDVA and CDVA values. A bioptic procedure could be planned for those cases in which the refractive sphere’s correction was required, and excimer ablation may compromise the visual quality or exceed its safety limits. 

Beyond these good short-term results, it is crucial to know these improvements remain stable over time. Consequently, long-term follow-up results are required. The studies that evaluated excimer laser for postoperative refractive error correction in post-DALK did not reach an extended follow-up. In our study, the mean post-Femto-LASIK follow-up period was 48.0 ± 12.6 months (ranging from 36 to 60 months). The visual, refractive, and keratometry outcomes remained stable in 8 of the 10 eyes over the follow-up (Table 1). In case 7, there was an increase in myopia over 60 months of follow-up and a decrease in UDVA. However, no changes in CDVA or keratometry reading were found. By contrast, in case 9, refractive error and UDVA instability were accompanied by significant changes in the Keratometry readings, which showed a refractive error regression. Notedly, the DALK indication for this case was RK, which implies that of four eyes in which the DALK indication was RK, one eye (25%) experienced a regression of the refractive cylinder. Oral et al. [35] found that in eyes with previous RK, the astigmatism correction with LASIK is difficult and tends to regress over time. Although our case series were eyes that underwent DALK surgery, RK incisions usually were made up to 90% of the corneal depth, implying that RK scars remain in the host cornea. These remaining scars could affect the long-term refractive outcomes of the DALK procedure, and the Femto-LASIK results in those cases might be less stable than for other DALK indications. It would be interesting to analyze the impact of corneal transplant indication on the DALK results. In line with this, Einan-Lifshitz et al. [36] reported that in eyes with prior RK, the success rate of big bubble (BB) creation was significantly lower than in patients with keratoconus or pathologies that do not involve the deep corneal layers. The authors pointed out that the manual lamellar dissection should be considered the primary surgical technique rather than the BB technique in post-RK eyes. These findings suggest that the indication of corneal transplant could influence the DALK results. However, further long-term studies would be required to assess whether the DALK indications affect refractive stability. 

In our case series, the DALK indication in five eyes was a corneal ectasia (keratoconus or post-LASIK ectasia). In all of them, the refractive error, UDVA, CDVA values, and K reading remained stable at their respective postoperative follow-up visits (ranging from 36 to 60 months). Furthermore, the pachymetric parameters studied (CCT, TCT) were stable over the follow-up period. Despite these encouraging outcomes, it is essential to be cautious because in the eyes that undergo DALK for keratoconus, there is a risk of ectasia after 15–20 years of DALK [37,38]. 

In the present study, no complications occurred during the Femto-LASIK procedure or throughout the follow-up period (including flap-related complications, graft failure, and corneal ectasia). We chose the femtosecond laser to create the flap as it can perform more predictable and uniform flap thickness and stromal bed and less intraoperative epithelial injury [39,40]. Furthermore, it allows for the performance of the excimer laser ablation within the corneal graft limits without involving the graft–host junction [14] (see Figure 1). Moreover, Femto-LASIK surgery does not significantly change the ECD [41], even in Post-DMEK eyes [42]. The ECD in this case series was stable over the post-Femto-LASIK surgery follow-up. Although there were no complications, further long-term prospective studies enrolling more patients should be performed to assess possible complications and the stability of the procedure.

In conclusion, our findings suggest that Femto-LASIK might safely and effectively correct residual astigmatism after DALK. Despite these encouraging results, it is essential to be cautious because of the small number of cases enrolled in this study. Further long-term studies, including a larger number of cases, are required to properly assess the stability of this surgery and confirm the safety of the procedure. The refractive stability in eyes with prior RK might be lower than for other DALK indications. 

## Figures and Tables

**Table 1:** Uncorrected (UDVA) and best-corrected visual acuity (CDVA) (expressed in decimal value) and refractive error before and after Femto-LASIK for each case.

				Follow-Up						
Case/Gender/Age	DALK Indication	Interval DALK-FL	Lens Status	Parameters	Pre-FL	6 Months	12 Months	36 Months	60 Months	Comments
# 1 (Female/ 54 years old)	Central keratoconus	32 months	Phakic	UDVA	0.05	0.2	0.2	0.2		
				CDVA	0.5	0.6	0.6	0.7		
				Refraction	−2.00–5.00 × 45°	−1.75–1.25 × 80°	−2.00–1.25 × 75°	−1.75–1.50 × 75°		
				K reading	43.25/50.00	42.50/45.00	43.00/45.50	43.00/45.50		
# 2 (Male/ 46 years old)	Paracentral keratoconus	27 months	Pseudophakic with monofocal IOL	UDVA	0.2	0.5	0.5	0.5		Bioptics: ICL 3 months after FL for hyperopia correction
				CDVA	0.6	0.6	0.7	0.7		
				Refraction	+3.50–4.50 × 170°	+0.50–0.50 × 105°	+0.50–0.75 × 105°	+0.50–1.00 × 100°		
				K reading	42.25/46.25	42.00/43.00	42.50/43.50	42.00/43.25		
# 3 (Male/ 45 years old)	Paracentral keratoconus	31 months	Phakic	UDVA	0.2	0.6	0.6	0.6	0.7	
				CDVA	0.7	0.8	0.8	0.8	0.9	
				Refraction	0.00–4.00 × 170°	−0.50–0.75 × 35°	−0.50–1.00 × 45°	0.00–1.00 × 40°	0.00–1.00 × 40°	
				K reading	43.75/47.50	44.25/45.50	44.00/45.00	44.50/45.00	44.25/45.45	
# 4 (Male/ 49 years old)	Post-LASIK ectasia	33 months	Phakic	UDVA	0.1	0.4	0.4	0.5	0.5	Bioptics: ICL 3 months after FL for myopia correction
				CDVA	0.5	0.6	0.6	0.7	0.7	
				Refraction	−3.50–5.00 × 5°	0.00–1.00 × 10°	0.00–1.50 × 15°	0.00–1.00 × 10°	0.00–1.00 × 10°	
				K reading	41.75/44.75	41.00/42.25	40.75/42.75	40.75/42.50	41.00/42.25	
# 5 (Male/ 45 years old)	Post-LASIK ectasia	21 months	Phakic	UDVA	0.1	0.6	0.7	0.7		
				CDVA	0.8	0.9	1.0	1.0		
				Refraction	0.00–4.75 × 100°	0.00–1.50 × 90	+0.50–1.75 × 95°	+0.50–1.75 × 95°		
				K reading	41.00/45.50	40.25/43.00	40.25/42.50	40.25/42.50		
# 6 (Male/ 52 years old)	Radial keratotomy	30 months	Phakic	UDVA	0.2	0.4	0.4	0.4		
				CDVA	0.9	0.9	1.0	1.0		
				Refraction	−2.50–2.50 × 160°	−2.25–0.50 × 180°	−2.00–0.50 × 180°	−2.00–0.50 × 180°		
				K reading	41.50/43.50	41.25/41.25	41.25/42.00	41.00/41.75		
# 7 (Female/ 47 years old)	Radial keratotomy	33 months	Pseudophakic with monofocal IOL	UDVA	0.1	0.4	0.4	0.3	0.2	
				CDVA	0.6	0.7	0.7	0.8	0.8	
				Refraction	0.00–2.75 × 90°	−0.75–0.75 × 75°	−1.50–1.00 × 90°	−1.75–0.50 × 80°	−2.00–0.50 × 90°	
				K reading	44.75/47.00	44.25/45.50	44.00/45.50	44.00/45.25	44.50/45.75	
# 8 (Female/ 54 years old)	Radial keratotomy	23 months	Pseudophakic with monofocal IOL	UDVA	0.1	0.5	0.5	0.6	0.6	
				CDVA	0.7	0.8	0.8	0.8	0.8	
				Refraction	0.00–3.50 × 160°	−0.50–1.00 × 80°	−0.50–1.00 × 80°	−0.50–0.75 × 85°	−0.50–0.75 × 90°	
				K reading	43.75/48.75	44.00/45.25	44.50/45.00	44.00/45.75	44.25/45.50	
# 9 (Male/ 52 years old)	Radial keratotomy	22 months	Pseudophakic with monofocal IOL	UDVA	0.16	0.7	0.6	0.6	0.4	Regression
				CDVA	0.5	0.8	0.7	0.9	0.9	
				Refraction	+0.50–2.50 × 60°	0.00–0.50 × 70°	+0.50–1.50 × 75°	+1.00–2.00 × 65°	+1.00–3.00 × 70°	
				K reading	42.00/44.50	42.50/43.50	42.25/44.00	41.75/45.00	41.50/45.00	
# 10 (Female/ 74 years old)	Leukoma	24 months	Pseudophakic with monofocal IOL	UDVA	0.1	0.4	0.4	0.5		
				CDVA	0.5	0.5	0.5	0.7		
				Refraction	0.00–4.25 × 40°	−0.00–1.50 × 100°	+0.50–1.00 × 130°	+0.50–0.50 × 140°		
				K reading	42.75/46.00	43.00/44.50	43.00/44.50	43.25/44.25		

DALK: deep anterior lamellar keratoplasty; FL: Femto-LASIK; K: keratometry; IOL: intraocular lens; ICL: implantable collamer lens.

## Data Availability

The data presented in this study are available on request from the corresponding author.
